# Effects of Job Demands and Resources on Positive and Negative Affect of Delivery Drivers in China

**DOI:** 10.3390/ijerph19138140

**Published:** 2022-07-02

**Authors:** Congcong Zhang, Sophie Sitar, Chien-Chung Huang

**Affiliations:** 1 Department of Youth Movement History, China Youth University of Political Studies, Beijing 100089, China; zhangcc@cyu.edu.cn; 2 Rutgers Law School, Newark, NJ 07102, USA; sjs437@scarletmail.rutgers.edu; 3 School of Social Work, Rutgers University, New Brunswick, NJ 08901, USA

**Keywords:** job demands, job resources, positive affect, negative affect, delivery driver, China

## Abstract

The delivery workforce in China has grown substantially within the past decade. As this industry has grown, job demands (JD) have also increased to ensure productivity. Accordingly, delivery drivers are increasingly facing volatile and stressful work conditions that could influence their Positive and Negative Affect (PANA), which has been an important predictor of their health and well-being. This study utilizes a sample of modern delivery drivers in Beijing, China (*n* = 240) to study how the job demands-resources theory (JD-R) was related to PANA. The results indicate delivery drivers experience relatively high JD and moderate JR at their places of employment. As expected, JR were positively associated with PA and negatively associated with NA. Meanwhile, JD were positively related to NA; however, JD were also positively associated with PA within the sample. Further interaction analysis showed that only drivers with high JD and high JR were positively associated with PA and that drivers with high JD and low JR were associated with high NA and had no effect on PA. These findings call for support for Chinese delivery drivers who face high job stress and high job demands.

## 1. Introduction

As the digital economy has rapidly grown, China has seen considerable growth in its delivery sector within the past decade [1,2,3]. In 2011, the online-to-offline food market was worth 22.2 billion yuan (USD $3.4 billion) [4], and by 2020, it totaled approximately 665 billion yuan (USD $103 billion) [5]. This increasing market led to the creation of more than 7 million food delivery driver jobs across the country by 2019 [6]. Meanwhile, in the package delivery industry, deliveries exceeded 60 billion, drawing in a revenue of over 700 billion yuan (USD $108.5 billion). This accounted for 7.6‰ of China’s 2019 GDP [3] (State Post Office, 2020). Notably, the number of Chinese package delivery drivers increased by 50% from 2016 to 2018. By 2018, the country had 3 million individuals employed as package delivery drivers [1]. However, these expanding labor markets have come with increasing work demands and work stress for delivery drivers [7,8,9,10,11]. Therefore, it should come as no surprise that delivery drivers experience fewer positive emotions and affect and more negative emotions and affect at their jobs [2,12]. Thus, it is critical to explore the effects of job demands (JD) and job resources (JR) on delivery drivers’ health and well-being to be better equipped to support them as this industry continues to boom.

First, to understand the significance JD and JR have on delivery drivers, it is critical to examine the positive and negative affect (PANA) delivery drivers experience. PANA are important indicators of well-being [13,14]. Positive affect (PA) refers to an individual’s propensity to feel positive emotions such as enthusiasm, confidence, or excitement, as well as positively interact with others and with life’s challenges. Individuals with self-reported high PA generally experience pleasant and enjoyable daily interactions. Meanwhile, negative affect (NA) indicates an individual’s inclination to feel negative affective states such as guilt, fear, or nervousness, and feel a more negative sense of interaction with the world [15]. Thus, PA and NA are influential to individual life experiences and can alter life events such as work performance, health, and general well-being [16,17,18,19]. However, minimal studies have researched the effects of job demands and resources theory (JD-R) on PANA for Chinese delivery drivers. Thus, this study endeavors to understand this relationship and to provide practical implications for intervention to mitigate NA and improve PA for vulnerable delivery drivers.

## 2. Development of Delivery Drivers in China

Delivery drivers often face significant work-related stress. Lin and Li [10] found that over 95% of food delivery drivers report experiencing occupational stress, and three-quarters report such high levels of stress that indicated high health risks. There is a multitude of reasons to explain occupation stress within this profession. First, studies show algorithmic management tools that monitor driver performance contribute to the growing labor intensity for delivery drivers and subsequently increase burnout and turnover rates amongst drivers [2,20]. For example, Zheng [11] found that 25% of food delivery drivers had already left their position at least once. Second, drivers frequently work long shifts, are demanded to have delivery efficiency, and are forced to engage in emotional labor when interacting with customers [12]. While some of these attributes are factored into the driver’s compensation, aspects such as dealing with emotional customers are generally not. The pressures of providing consistent “service with a smile” without necessarily receiving compensation for the performance can exhaust drivers and lead to burnout. Third, workers’ minimum earnings are not guaranteed, as the demand and supply of delivery drivers change frequently. When the demand for food delivery is low, demand for delivery drivers becomes low, which can cause some drivers to struggle financially, which adds undue stress [2]. Fourth, the COVID-19 pandemic has further exacerbated these poor work conditions by imposing increased health risks, job insecurity, and financial instability for many delivery drivers [9].

As with food delivery drivers, package delivery drivers also experience significant job demands and burnout. Reports indicate that over half of all package delivery drivers work more than 10 h a day, and around one-fifth work more than 12 h a day [1,7]. As a result, many package delivery drivers struggle with work-life balance, as there is considerably less time for rest and leisure [8]. It follows that package delivery drivers report low job satisfaction and life well-being, as well as high occupational stress, burnout, and turnover [21,22,23,24].

Moreover, for many delivery drivers, there are minimal other economic opportunities, and thus, many feel stuck within this industry. For example, Zheng [11] found that 17% of package delivery drivers had left their position at least once; however, many who returned were forced back to their job due to a lack of work opportunities elsewhere. First, there are simply not enough jobs available to meet the demand. Second, many delivery drivers are migrant workers with low educational attainment and thus lack the prerequisite qualifications necessary for upward mobility [8,25]. Therefore, many delivery drivers who make good faith efforts to leave the industry are forced to come back, often unwillingly. To prevent these cycles from perpetuating, further investigation is required to discover protective factors that can mitigate burnout in the large and vulnerable Chinese labor force.

## 3. Positive Affect and Negative Affect (PANA) and Its Relations with Work-Life

PANA are not only physiological affects but are also important indicators of possible life outcomes [14,17]. Positive affect (PA) can give rise to individual success, health, and happiness, while negative affect (NA) can inhibit or thwart such ambitions. Accordingly, PA presents positive individual characteristics such as confidence, optimism, sociability, effective coping, and flexibility which encourage a person to seek out help when needed as opposed to retreating to isolation. In addition, Fredrickson’s broaden-and-build theory [26] postulates these positive emotions stemming from PA can expand an individual’s behavioral or emotional repertoire, which help the individual accrue and learn new skills. Thus, the individual is better equipped to endure physical and psychological stress more easily than those without PA. Additionally, other broaden-and-build-focused studies have found PA within a work environment is positively associated with work engagement and performance, effective coping strategies, job satisfaction, and emotional intelligence [19,27,28]. Finally, PA amongst employees can also significantly reduce emotions and stress symptoms, turnover intentions, maladaptive coping, and depression and anxiety [18,28].

By contrast, NA involves attributes that can inhibit success and happiness and lead to isolation [13,29]. NA presents negative individual characteristics such as guilt, anxiety, rumination, and fear. NA is also associated with emotional dysregulation and psychiatric symptoms [16,30], as well as health conditions such as hypertension [31]. Individuals, especially within the work context, are more likely to focus and look for negative environment cues that contradict their psychological contract, which is a reciprocal exchange between an individual and another party [29]. In addition, Weiss and Cropanzano’s affective events theory [32] explains how employees’ internal influences (i.e., emotions, moods, or mental states) can positively or negatively affect aspects of their work environment such as work performance, organizational commitment, and job satisfaction. Thus, NA, for example, is foretelling of workplace deviance such as absenteeism, theft, poor job performance, and general poor health of employees [33,34].

PANA is an influential factor of individual success and well-being within the work environment [35,36]. There have been numerous studies supporting the significance of PANA, but none have examined the relationship between JD-R and PANA amongst delivery drivers in China. Thus, this study seeks to explore any such relationship and utilize the findings to shed light on ways to improve occupational stress amongst Chinese delivery drivers.

## 4. The Job Demands-Resources (JD-R) Theory and Research Hypotheses

The JD-R theory is a theoretical framework that seeks to explain how various work conditions, positive or negative, can affect employees’ health and job performance [37,38]. First, it divides working conditions into job demands (JD) or job resources (JR) categories. JD are conditions that require an individual to sustain prolonged physical and/or mental efforts despite potential physiological and psychological costs, such as exhaustion. Alternatively, JR are conditions that help alleviate the physiological and psychological costs imposed by JD and encourage work-based achievement. Demerouti et al. [39] posits poor JD-R affect work and well-being via two processes: (1) demanding aspects of work tasks leads to exhaustion and burnout and (2) subsequent lack of resources makes fulfilling increasing JD more difficult and thus exacerbates worker withdrawal, disengagement, and burnout.

JD-R theory has been validated by various studies which have examined different occupational groups [37,40,41] as well as health and work-related outcome variables, such as psychological distress and mental health [42,43,44,45,46]. In addition, cross-cultural studies have used the JD-R model to discover JD and JR affect employees and their ability to perform at work. JD deplete energy, which causes fatigue. However, if available, JR can motivate workers, alleviate negative effects of JD, and reenergize workers to maintain productive and successful at work, but when JR are low, the negative effects of JD can become compounded beyond repair [47,48,49,50,51].

Though the JD-R literature is well established, empirical JD-R studies on PANA and in the delivery driver sector specifically are limited. Given the dramatic increase in the delivery industry in China, there is a research gap in the JD-R and PANA of delivery drivers. The aim of this study is to investigate the extent of the JD-R theory applies to PANA of delivery drivers in China. Based on the JD-R theory, the broaden-and-build theory, and the affective events theory, the following research hypotheses involving JD-R and PANA were proposed:

**Hypothesis** **1** **(H1).** *JD reduce PA and increase NA*.

**Hypothesis** **2** **(H2).** *JR increase PA and reduce NA*.

**Hypothesis** **3** **(H3).** *JR moderates the relationship between JD and PANA. The higher the level of JR, the weaker the relationship is between JD and PANA*.

## 5. Methods

### 5.1. Data and Sample

The data was collected via internet-based surveys administered to food and package delivery drivers in Beijing, China. First, food delivery respondents were recruited via convenience sampling. The first author engaged with approximately 300 food delivery drivers near large shopping malls with significant concentrations of dining establishments between 11 June and 30 June 2021. Each food delivery driver was messaged a link to access the internet-based survey. Of those sent the link, 110 food delivery drivers participated. These drivers were employed by either Meituan, Eleme, Dada, or Shansong food delivery platforms. After omitting 4 cases with incomplete answers, the final analytic sample of food delivery drivers consisted of 106 respondents.

Second, we recruited different package delivery drivers who worked at one of the main Beijing package distribution centers. Of all distribution centers, fifteen centers, located in the Haidian, Chaoyang, Fengtai and Daxing Districts of Beijing, were selected to choose drivers from. These centers housed companies such as S.F. Express, STO Express, YTO Express, ZTO Express, YunDa Express, and Jingdong Logistics. On 6 July 2021, we sent the survey link to package delivery drivers that were employed with these chosen distribution centers. Approximately 260 drivers received the link. Participants were sent a reminder to participate 7 and 14 days after the original invitation was sent out. By 31 August 2021, 145 package delivery drivers had completed the survey. After excluding 11 incomplete surveys, the final analytic sample was 134 package delivery drivers. The final analytic sample of this study, including both food and package delivery drivers, is 240 (106 + 134) drivers.

The drivers, on average, took about 12 min to complete the survey. Each participant was notified of their anonymity within the study, given an informed consent notice, and informed of their voluntary participation within the study. Those who completed the survey received 5 RMB (1 USD). Additionally, this research protocol was approved by the research review committee at the Huamin Research Center at Rutgers University and China Youth University of Political Studies.

### 5.2. Measures

We used the short form version of the International Positive and Negative Affect Schedule (I-PANAS-SF [52]), which is a 10-item scale that has demonstrated high reliability and validity, to measure PANA [53,54]. This version of the I-PANAS-SF requires participants to self-report the frequency they experienced feeling upset, inspired, determined, and other certain emotions within the past two-week period. Possible answers ranged from 1 (“never”) to 5 (“always”). We then took the items that coincided with PA and NA and averaged the scores. Here, the Cronbach’s alpha of PANA scale was 0.78.

We used Lequeurre et al.’s Questionnaire sur les Ressources et Contraintes Profesionnelles (QRCP) [55] to measure the explanatory JD and JR variables Chinese delivery drivers’ experience. First, JD were measured by adapting items from workload and emotional workload in QCRP. The QCRP measures workload as the intersection between time needed to meet work responsibilities and the actual amount of time employees are given to complete the task. Meanwhile, emotional workload describes the effort needed to cope with job-inherent emotions. For example, delivery drivers must remain calm and efficient even when experiencing pressures from challenging customers.

Second, JR were similarly measured by assessing relationships with colleagues, relationships with supervisors, and support received from the company and customers. Relationships with both colleagues and supervisors describe the extent to which an individual perceives that they can receive social support from their co-worker and from their supervisor, respectively. The last subconstruct, support from company and customers, examines how much support participants feel they receive from their company and their customers when challenges occur or deliveries are late. JD and JR were each measured with 4 items. Response categories followed a 7-point Likert scale, which ranged from 1 (“never”) to 7 (“always”). Participants with higher scores are interpreted as having greater JD or JR at their job. In addition, the mean score of JD scores and JR scores were calculated as the average of all corresponding item responses. Here, the Cronbach’s alpha for the adapted QRCP items was 0.82.

To account for various demographic and socioeconomic characteristics that may affect our variables of interest, we collected data on sex (female = 0, male = 1), age, educational attainment (below high school; high school; and above high school), marital status (0 = married, 1 = never married), and type of delivery (0 = food, 1 = package). Full sample demographics are displayed in Table 1. A total of 80% of the participants identified as male. The average age of participants was 35.5. Most respondents had either below high school education (42.9%) or held a high school degree (33.3%). A total of 42% had never married, and 55.8% delivered packages rather than food.

## 6. Analytical Approach

First, we performed descriptive and Pearson’s correlation analyses to observe the sample characteristics and the correlations amongst all variables. Then, we conducted ordinary least squares (OLS) regression analysis to estimate the effects of JD-R on PANA, controlling for demographic and socioeconomic characteristics. The common method variance analysis was performed, and the results showed that only 22.4% of the variance shared by JD-R and PANA items, suggesting common method variance was not an issue in the data. The normality tests of the variables were performance. The skewness values ranged from 0.11 (job demands) to 0.92 (job resources) while the kurtosis values were between 0.16 (job demands) and 0.69 (PA). All analyses were conducted using Stata software 16.0.

## 7. Results

Table 1 presents the descriptive statistics of the variables. The sample reported high PA (M = 3.2) and moderate NA (M = 2.9). They also reported comparatively high JD (M = 4.9) and moderate JR (M = 4.3). The Pearson’s correlation analysis, as shown in Table 2, indicated a positive association between JD and PA (r = 0.25, *p* < 0.001) and NA (r = 0.19, *p* < 0.001), while JR was positively associated with PA (r = 0.24, *p* < 0.001). In addition, PA and NA had a significant correlation with each other (r = 0.41, *p* < 0.001). JD and JR also had a highly positive correlation with one another (r = 0.30, *p* < 0.001). JD was positively correlated with package delivery drivers, male gender, and drivers with high school education, while it was negatively related to age and to drivers with above high school education. JR tended to be higher for drivers with younger ages and with high school educations.

Table 3 presents the standardized estimates of PANA from OLS regression. Both JD and JR had a positive and strong association with PA (Beta = 0.23 and 0.19, *p* < 0.001). However, JD was positively associated with NA (Beta = 0.28, *p* < 0.001), while JR had no effect on NA. Compared to drivers with above high school education, drivers with only high school education had significantly low NA (Beta = −0.21, *p* < 0.001). These findings are partial support for Hypotheses 1 and 2.

We used two interaction specifications to assess the buffer effect of JR on JD. One is a linear interaction (JD*JR), and one is a category interaction (Low JD and JR, high JD low JR, high JR low JD, and high JD and JR). We inserted the interaction variable and Table 3 variables into a regression analysis, and Table 4 presents these results. For simplicity, we only presented interaction results on JD and JR. The results for other variables were like the ones in Table 3. The linear interaction, along with the categorical specification, showed that drivers with high JD and high JR had significantly higher PA. The further binary tests of estimates between categories, as shown in the bottom panel of Table 4, showed that the drivers with high JD and JR appeared to have high PA compared to the drivers in the other three categories. The linear interaction between JD and JR, however, did not have effect on NA. However, the categorical interaction showed that drivers with high JD and low JR had significantly high NR (Beta = 0.21, *p* < 0.05) compared to drivers with low JD and JR. The further binary tests of estimates between categories showed that drivers with high JD and low JR appear to have high NA compared to the other three categories. The findings confirm Hypothesis 3.

## 8. Discussion

The descriptive results show the sample of delivery drivers within the study experience relatively high JD and moderate JR in their work. The drivers also report high PA and moderate NA. These regression results support our hypothesis that JD-R were related to PANA for Chinese delivery drivers. Specifically, high JD were associated with high NA, which indicates there is an underlying energy depletion process present amongst drivers. Essentially, this means the high JD are inducing delivery drivers to experience symptoms such as fatigue, energy depletion, stress, and/or burnout [56]. Additionally, JR were positively associated with high PA and low NA, which indicates an underlying motivational process that was helping drivers build energy and skills to succeed at work. The regression findings also show that JD, per se, have positive effect on PA in the delivery drivers in China. This may be due to the fact that high JD are associated with high income for delivery drivers, as their income is determined by the quantity of delivery they made, and high income may be associated with high PA. Further research is warranted to examine the relationships between JD, income, and PA in Chinese delivery drivers.

The interaction analysis of JD and JR on PANA showed that JR may have a buffer effect between JD and PANA. Drivers with high JD and high JR had significantly higher PA compared to other drivers. In addition, drivers with high JD and low JR had significantly high NR compared to other drivers. These findings emphasize how important it is to have supportive JR to buffer the effects of JD on PANA for Chinese delivery drivers. Together, these findings are consistent with and expand upon previous findings of the relationship between JD-R and various work-related organizations [47,48,50,51]. The results show JD-R, the underlying energy depletion, and motivational processes, are significant predictors of PANA.

## 9. Implications and Limitations

This study has theoretical, research, and practice implications. This study further extends the theoretical development and application of JD-R model in Chinese delivery drivers’ PANA. The study enhances the knowledge of JD, JR, and PANA as vital intercultural construct by providing support for the relationships between JD, JR, and PANA in delivery drivers in China.

Regarding research implications for future studies, as JD-R were associated with PANA of delivery drivers in China, a natural extension in this line of research is to examine how the effects of JD-R and PANA on job performance of Chinese delivery drivers. If PANA mediated the effects between JD-R and job performance, employers could use PANA as intervention target for improving job performance. In addition, as JD was positively related to PA, further research is warranted to distinguish the relations among JD, earnings, PA, and job performance.

With respect to practice implications, the participants reported high JD, as well as moderate JR at their disposal. Given the consequences high JD can impose and its positive predictability of NA, employers of Chinese delivery drivers create efforts to reduce JD while increasing JR to mitigate NA. Given the importance of JR’s buffering effects on JD, employers and coworkers need to promote a friendly and supportive work environment for their drivers to reduce risk of burnout and turnover amongst drivers [2,12,20]. Also, employers may utilize PANA as a key point of intervention for high JD employers by implementing several interventions and services to support PA and reduce NA. For example, employers can consider implementing mindfulness-based services that have shown effectively improve PA and reduce NA in employees [57,58,59].

Additionally, our study is one of few to center on the work experiences of food and package delivery drivers in China, but these findings must be contextualized within the study’s limitations. First, we used cross-sectional data, which only allowed us to approximate associative relationships amongst the main variables. Causal relations cannot be established. Second, the findings might be subjected to omitted variable bias because our model may not have included all variables that may affect JD-R, burnout, and mindfulness. Third, the results may have been subject to bias and reporting errors due to the reliance on self-reporting data collection. Fourth, the study sample was recruited via convenience sampling, which limits the generalizability of the results. A future study might consider using random sampling instead to achieve stronger results.

## 10. Conclusions

Consistent with the JD-R theory, our results confirm JD and JR were associated with delivery drivers’ PANA in China. In addition, the results contribute to past research of how JD-R can be related to the general well-being of several differing work-related agencies/individuals. Delivery drivers are a vulnerable occupational group, particularly because of high job demands and low job resources. The finding that JR have a significant buffering effect on JD and PANA calls for intervention and services to promote more supportive JR for delivery drivers with high JD.

## Figures and Tables

**Table 1:** Descriptive Statistics of Key Variables.

	Mean (S.D.)
1. Positive Affect [1–5]	3.2 (0.9)
2. Negative Affect [1–5]	2.9 (0.9)
2. Job Demands [1–7]	4.9 (1.3)
3. Job Resources [1–7]	4.3 (1.4)
4. Type of Delivery [%]	
Food Delivery Driver	44.2
Package Delivery Driver	55.8
5. Male [%]	79.6
6. Age [18–60]	35.5 (10.9)
7. Education [%]	
Below High School	42.9
High School or above	57.1
Above High School	23.8
8. Marital Status [%]	
Never Married	42.1
Married	57.9

Note: *n* = 240. Numbers in brackets show ranges of the variables.

**Table 2:** Correlation Analysis of Key Variables.

	1	2	3	4	5	6	7	8	9	10
1. Positive Affect	-									
2. Negative Affect	0.41 ***	-								
3. Job Demands	0.25 ***	0.19 **	-							
4. Job Resources	0.24 ***	−0.03	0.30 ***	-						
5. Package Delivery Driver	0.08	0.09	0.29 ***	0.09	-					
6. Male	0.06	−0.01	0.22 ***	0.16	0.13 *	-				
7. Age	−0.06	−0.01	−0.33 ***	−0.15 *	−0.36 ***	−0.21 **	-			
8. Education—Below High School	0.02	0.05	−0.06	−0.05	0.04	0.00	0.07	-		
9. Education—High School	0.01	−0.09	0.32 ***	0.13 *	0.17 **	0.10	−0.22 ***	−0.61 ***	-	
10. Education—Above High School	−0.02	0.05	−0.27 ***	−0.09	−0.23 ***	−0.11	0.16 *	−0.48 ***	−0.39 ***	-
11. Never Married	−0.03	0.02	−0.01	0.01	0.01	0.08	0.02	−0.16 *	0.02	0.16 *

Note: *n* = 240. * *p* < 0.05, ** *p* < 0.01, *** *p* < 0.001.

**Table 3:** Regression Analysis of Positive and Negative Affect.

	Positive Affect			Negative Affect		
	Beta	S. E.	*p*	Beta	S. E.	*p*
Job Demands	0.23	0.05	**	0.28	0.05	***
Job Resources	0.19	0.04	**	−0.08	0.04	
Package Delivery Driver	0.03	0.12		0.08	0.14	
Male	−0.01	0.14		−0.03	0.16	
Age	0.03	0.01		0.07	0.01	
Education—Below High School	−0.03	0.15		−0.07	0.16	
Education—High School	−0.11	0.16		−0.21	0.18	*
Never Married	−0.02	0.12		0.03	0.13	
Adjusted R-square	0.07			0.05		

Note: *n* = 240. * *p* < 0.05, ** *p* < 0.01, *** *p* < 0.001.

**Table 4:** Interaction Analysis of JD and JR on PANA.

	Positive Affect			Negative Affect		
	Beta	S. E.	*p*	Beta	S. E.	*p*
Specification 1						
Job Demands	−0.07	0.10		0.36	0.11	*
Job Resources	−0.29	0.14		0.04	0.15	
Job Demands * Job Resources	0.66	0.02	*	−0.17	0.03	
Specification 2						
Low Job Demands, Low Job Resources [1]	-	-		-	-	
High Job Demands, Low Job Resources [2]	−0.01	0.21		0.21	0.23	*
Low Job Demands, High Job Resources [3]	0.01	0.16		0.06	0.18	
High Job Demands, High Job Resources [4]	0.31	0.17	**	0.08	0.19	
Binary Tests:						
[2] = [3]		0.03			3.50	*
[2] = [4]		8.32	***		3.60	*
[3] = [4]		7.84	***		0.40	

Note: *n* = 240. * *p* < 0.05, ** *p* < 0.01, *** *p* < 0.001.

## Data Availability

Data available on request due to privacy restrictions.
